# Using the Ethaline Electropolishing Method on the Internal Surface of Additive Manufactured Tubes

**DOI:** 10.3390/ma17194915

**Published:** 2024-10-08

**Authors:** Dongyi Zou, Chaojiang Li, Yuxin Yang, Xin Jin, Shenggui Liu, Hongyi Zhang, Na Zhang

**Affiliations:** 1 School of Mechanical Engineering, Beijing Institute of Technology, Beijing 100081, China; zoudy0610@163.com (D.Z.); mecjli@bit.edu.cn (C.L.); yyxxhzlj@gmail.com (Y.Y.); liusg_email@163.com (S.L.);; 2 Department of Electronic and Computer Engineering, University of Alberta, 9120 116 St NW, Edmonton, AB T6G 1C9, Canada

**Keywords:** non-traditional machining, electropolishing, additive manufactured metal parts, deep eutectic solvents

## Abstract

Electropolishing is a widely used technique for polishing additive manufactured (AM) components, while complex internal surface polishing remains a challenge. In this study, we explore the use of ethaline as an electrolyte and investigate the effects of temperature, time, stirring speed, and voltage on the electropolishing effectiveness for AM tubes without pre-treatment through orthogonal experiments. The optimal combination of these factors is then applied in further electropolishing experiments on straight tubes with large length-to-diameter ratios and an angled tube. Our results indicate that temperature has the most significant impact on internal surface electropolishing performance, and other factors’ effects are also analyzed. Ethaline can be a promising electrolyte for internal surface electropolishing of AM components because of its high viscosity, which is validated by flow field simulation of the hydrodynamic conditions inside the tubes.

## 1. Introduction

Additive manufacturing (AM, also known as 3D printing or rapid prototyping) is recognized as a key strategy for manufacturing parts with complex structures. Selective laser melting (SLM) is a state-of-the-art AM process that can produce higher specific mechanical properties by sintering metallic powder with negligible void content [1]. However, metal parts produced by SLM often have high surface roughness. Defects such as the step effect, the balling effect, and powder adhesion [2,3,4] limit further application of SLM in aviation, aerospace, and other fields. The balling effect may lead to turbulence in fluid flow or stress concentration points, while powder adhesion may result in contamination of the internal surface, affecting the tubes’ properties such as corrosion resistance. In critical applications, surface defects can reduce performance over time, causing pressure losses and increased vulnerability to environmental factors [5]. For engine parts with inadequately polished internal walls, particles may adhere to the surface. Under harsh conditions, these particles can detach and enter the inner chamber, posing a risk to the engine.

Various surface finishing techniques are used to reduce surface roughness, including composite and non-traditional polishing methods. Reza et al. [6] proposed and experimentally validated effective process parameters in sequential processes to control surface topography and roughness, attaining surface quality with an arithmetic roughness value of 0.448 μm. Bai Yuchao et al. [7] used dry mechanical–electrochemical polishing to finish selective laser melted surfaces, reducing surface roughness from 8.72 μm to 0.75 μm. Wang Qinghua et al. [8] used pulsed laser micro-polishing to reduce the surface roughness (Sa) of the micro-end milled Ti-6Al-4V surface from 84 nm to 37 nm, an improvement of approximately 56%. Nevertheless, conventional mechanical machining is no longer suitable in terms of cost and time for additive manufactured structures which may present inner channels [9]. Among non-traditional surface finishing processes such as laser polishing which often generate heat-affected zones, reducing the surface consistency, electropolishing is a promising alternative because of its ability to treat inaccessible areas [10]. Zhao et al. [11] developed a novel electropolishing process to eliminate the adhesive powders and band protrusions generated during the laser melting process, and the surface roughness (Ra) of straight holes was reduced from 14.151 to 6.287 μm. Z. Chaghazardi et al. [12] investigated the electropolishing process of the internal walls of stainless steel 316 tubing considering potentials and times, and achieved an improvement of 60–80% for tubes with various lengths. The authors suggested that the interelectrode gap had a rather low impact on the final roughness but strongly impacted the final brightness.

The effectiveness of electrochemical polishing is influenced by many parameters, and the extent to which each parameter affects the final result varies. Therefore, many researchers have used the orthogonal experimental method to optimize the combination of these parameters. Prihandana GS et al. [13] optimized parameters through the Taguchi L9 orthogonal array to increase the material removal rate. Among the parameters, the electrolytes play a crucial role in controlling the material removal rate and preventing undesirable defects, which are typical directions in optimizing the electropolishing process [14]. A number of studies have investigated different kinds of electrolytes’ performance on the electropolishing process, including acidic and neutral electrolytes [15,16,17,18]. The most frequently used water-based electrolytes contain highly concentrated acids and other additives which may be highly toxic and dangerous to people. In addition, for specific materials, the application of aqueous electrolytes may increase the risk of hydrogen embrittlement. Hence, non-aqueous electrolytes such as deep eutectic solvents (DESs) are applied to the electropolishing process [9]. DESs are a type of solvent formed from a mixture of two or more compounds that, when combined, have a melting point much lower than that of either of the individual components. In this study, ethaline, one kind of DES, which is a mixture of choline chloride and ethylene glycol, is introduced as the electrolyte because of its negligible gas evolution at the anode/solution interface and high current efficiency [19]. However, many studies which use DESs as electrolytes focus on the external surface of the metal components.

Therefore, the main roadblock in internal surface electropolishing using ethaline is the lack of optimization method of the processing parameters combination. The factors affecting electropolishing of the internal surface by ethaline remain unclear. In this study, an electrochemical simulation of rough surfaces is conducted to show the changing trend of the surface topography using ethaline. Then, electrochemical experiments based on the orthogonal experimental design are conducted. The range analysis reveals the main parameter which has the greatest impact on electropolishing quality (i.e., Ra in this study). The surface roughness is measured, and Tafel curves are investigated to figure out the corrosion resistance change of the internal surface. Moreover, flow field simulations of ethaline and NaCl solution are conducted, and the streamline charts are compared to validate the impact of hydrodynamic conditions on electropolishing. The present work aims to investigate the key factor in ethaline electropolishing of internal surfaces and validate the viscosity’s impact on the electropolishing process in internal spaces.

## 2. Experiment and Simulation

### 2.1. Material Preparation

Samples with various lengths and angled tubes are prepared by SLM with an FS271M printer (Farsoon, Hunan, China). The corresponding manufacturing parameters are listed in Table 1. The diameter of the platinum electrodes is 1 mm.

**Figure 1:**
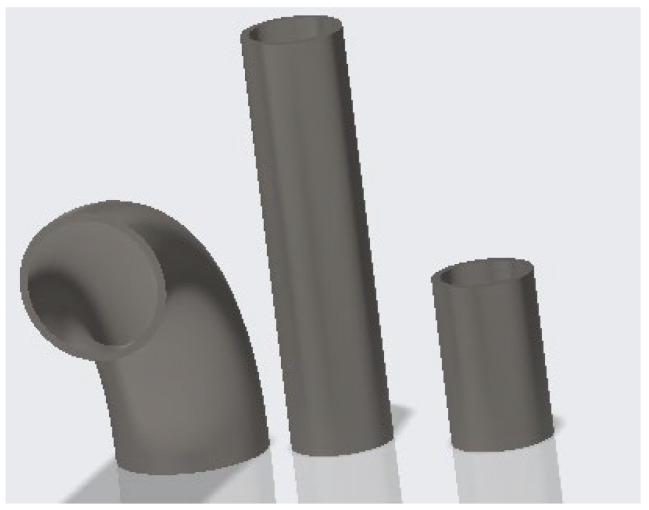
Three-dimensional models of tubes.

The 3D models of the tubes are shown in Figure 1. The detailed size parameters are shown in Table 2. The angled tube’s diameter is 8 mm and its bending radius is 13 mm. There are two kinds of straight tubes which are polished. The short one has a length of 10 mm, while the other has a length of 25 mm.

The ethaline is prepared by using glycol and choline chloride with a ratio of 2:1. The solutes are both from Sinopharm Chemical Reagent Co., Ltd. (Shanghai, China).

### 2.2. Simulation

**Figure 2:**
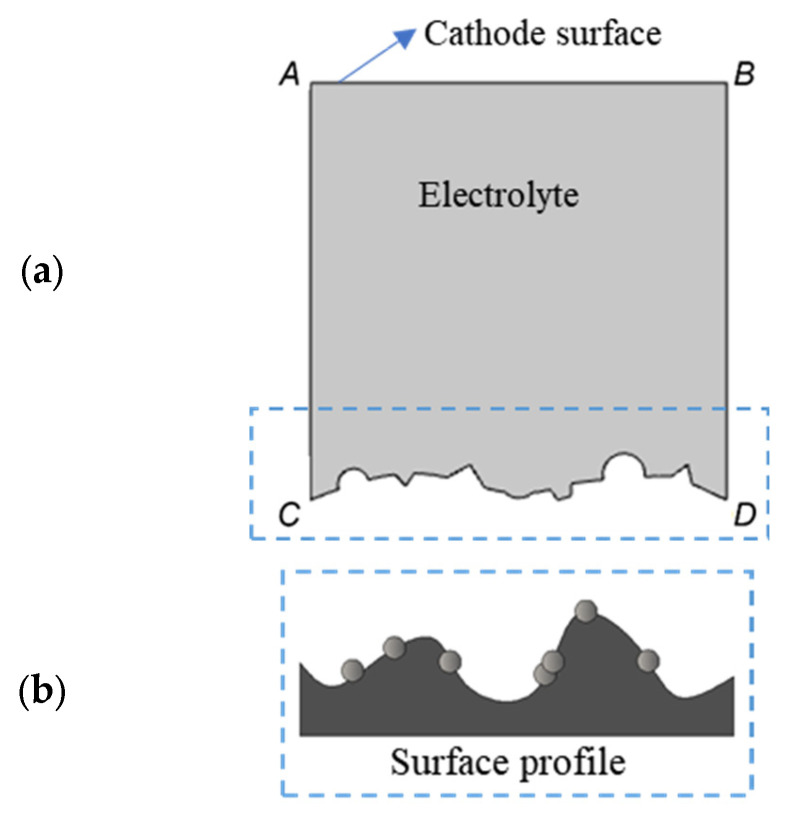
(**a**) Simplified simulation model; (**b**) partially melted metal particles sticking to as-printed side surface.

In this section, we emphasize the qualitative changes in the electrochemical process rather than the quantitative changes. Therefore, a simplified two-dimensional illustration in which the rough surfaces of both the cathode and the component are represented by simplified linear outlines is introduced to simulate the side surface of the three-dimensional printed components. As shown in Figure 2, lines AC and BD are the boundaries of the electrolyte, and line AB represents the cathode, while line CD represents the rough surface of the anode which includes protrusions and craters, alongside distinctive defects like adhered spherical particles specific to additive manufactured components. The gray region surrounded by these lines represents the electrolyte.

In addition, the boundaries AC and BD are set to be insulated, while the boundaries AB and CD are treated as equipotential surfaces due to their good conductivity. The boundary potentials are expressed as follows:

(1) $$\left.\Phi \right|\Gamma_{AB}=0,$$

(2) $$\left.\Phi \right|\Gamma_{CD}=\Phi ,$$

(3) $$\left.\frac{𝜕\phi }{𝜕n}\right|\Gamma_{AC,BD}=0$$

Equations (1) and (2) define the potentials of the cathode and anode, respectively. In the simulation, the cathode potential is set to 0, while the anode potential is set to the polishing voltage. Equation (3) indicates that the potential at the electrolyte boundary is 0.

The length of AB, AC, and BD is 9 mm. The material of the anode is 316 L stainless steel, and the material of the cathode is platinum. The voltage applied in the simulation is 7 V (i.e., Φ in Equation (2) is 7 V), and the conductivity of the electrolyte is 14 S/m with a density of 1.085 kg/m^3^. 

The electrochemical model in COMSOL is a pre-defined multi-physics model which consists of various current diffusion interfaces and deformed geometry interfaces. One corrosion interface which combines the Nernst–Planck interface, tertiary current distribution interface electroneutrality, and the deformed geometry formulation is selected in this work. The tertiary current distribution solves the full Nernst–Planck equation, describing the mass transport of each species through diffusion, migration, and convection, while adhering to the assumption of electroneutrality.

(4) $$N_{i}=-D_{i}𝛻c_{i}-z_{i}u_{m,i}Fc_{i}𝛻\phi_{l}+c_{i}u$$

where:

$D_{i}$ is the diffusion coefficient and its value in the simulation is $1\times 10^{-9}m^{2}/s$;

F is the Faraday constant;

R is the gas constant;

T is the temperature and is set to the polishing temperature;

$z_{i}$ is the valence state of the compounds in the reaction. 

According to the reaction, the main elements involved in 316 L dissolving are Fe→Fe^2+^ + 2e^−^, and the number of charges migrating from the electric field is 2. 

$c_{i}$ is the concentration of species i;

$N_{i}$ is the flux of species i;

u is the velocity of the fluid (for convection).

The current density expression is shown as follows:

(5) $$\mathrm{i}=-\frac{F^{2}}{RT}𝛻\phi \sum D_{i}C_{i}z_{i}^{2}-F\Sigma z_{i}D\mathrm{i}{𝛻}_{c_{i}}+vF\Sigma z_{i}c_{i},$$

Since any solution should maintain electroneutrality, the expression can be simplified.

(6) $$\mathrm{i}=-\frac{F^{2}}{RT}𝛻\phi \sum D_{i}C_{i}z_{i}^{2}-F\Sigma z_{i}D\mathrm{i}{𝛻}_{c_{i}}$$

**Figure 3:**
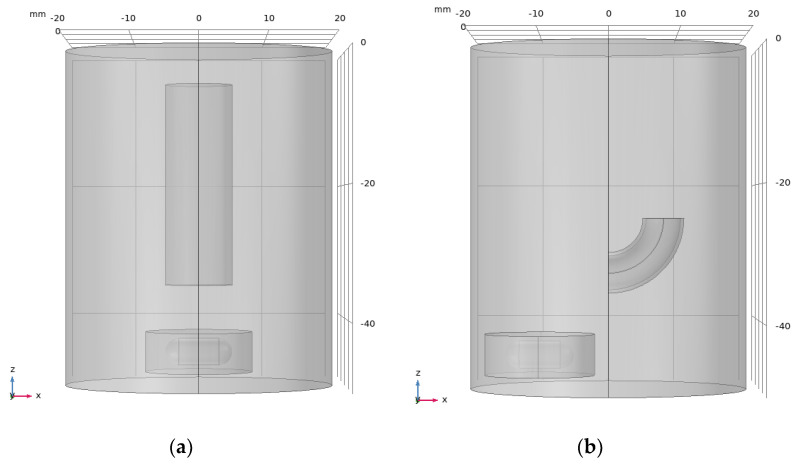
(**a**) Straight tube; (**b**) angled tube.

In this work, a flow field simulation is conducted by using the mixture model in COMSOL, and a frozen rotor is introduced in this work. The model is shown in Figure 3, and the main parameters for the flow field simulation are listed in Table 3.

### 2.3. Experiment Setup

**Figure 4:**
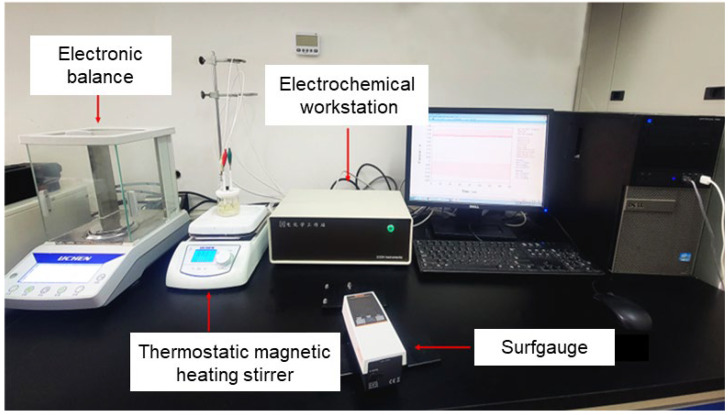
Experiment setup.

Figure 4 shows the experimental setup for the ECP process. An electrochemical workstation (CH Instruments Inc. (Austin, TX, USA)) was used to apply current between the electrodes. A beaker served as the reaction vessel which was filled with electrolytes and placed on the thermostatic magnetic heating stirrer. A surface roughness tester (Mitutoyo Measuring Instruments (Shanghai) Co., Ltd. (Shanghai, China)) was used to measure the surface roughness of the samples before and after polishing. Each time, the surface roughness was measured with a sampling length 0.8 mm and the evaluation length was 4 mm. The measurements were taken in both the horizontal and vertical directions of the sample, and the average value was calculated.

The experimental steps are detailed as follows: First, the electrolyte was prepared by using choline chloride and ethylene glycol with a molar ratio of 1:2.50 mL; ethaline was used in the experiments. Then, the samples were cleaned by ultrasonic cleaning in ethanol for 5 min and then in deionized water for 5 min. After ultrasonic cleaning, the samples were dried. Since there are many parameters that may have a potential impact on the polishing quality, orthogonal experiments were arranged to achieve the best combination of the parameters with the fewest tests. The optimal parameters were applied in the long tube and angled tube polishing experiments.

Temperature, polishing time, stirring speed, and voltage are the four main factors in the orthogonal experiments. The orthogonal array in this study is $L_{9}(3^{4})$. The levels are shown in Table 4.

Electrochemical testing was conducted by using an electrochemical workstation and based on the three-electrode system. The working electrode is the 316 L stainless steel samples, while the counter electrode is a platinum electrode. The reference electrode is composed of an Ag/AgCl electrode and KCl. Tafel curves are used to evaluate the corrosion resistance of the samples. The scanning range is from −1 V to 1 V, and the scanning rate is 0.005 V/s. 

In tube polishing, range analysis is used to find out the influential sequence of parameters that have an impact on polishing quality. Tafel curves are also used in this part to identify the corrosion resistance of the tubes.

## 3. Results and Discussion

### 3.1. Electrochemical Simulation Results

**Figure 5:**
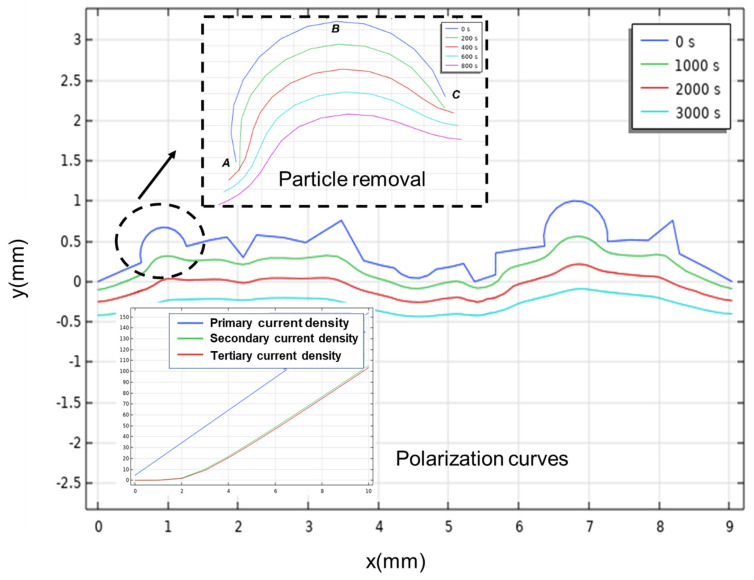
Plot of anode surface over time in tertiary current density distribution interface.

The surface profile changes over time in the electrochemical simulation are shown in Figure 5. In the first 1000 s, most of the sharp characteristics are removed due to electrochemical corrosion and transform into smoother bulges, but the adhered particles still retain their distinctive morphology. The particle removal thumbnail shows the morphological changes of the adhered particles in the initial 800 s. After another 2000 s, the bulges are almost removed but the concavity still exists, which indicates that the edge effect has the main impact on the removal of the adhered particles. Moreover, another two interfaces using different current density distributions are used in the simulation. According to the polarization curve thumbnail, a linear polarization curve for the primary current density distribution interface is found because the kinetics of the electrode process and the effect of concentration dependence are neglected. The polarization curves of another two interfaces follow a similar trend but the current in the tertiary current density distribution interface is slightly smaller than the current in the other one due to concentration polarization. A passivation region is found and it follows a similar trend as the polarization curves for the ALM (additive layer manufactured) 316 L samples in choline chloride–ethylene glycol. However, the current density plateau is not shown in the simulation result, and the key factors affecting the electropolishing effect on the internal surface of the tubes remain unclear. Therefore, an orthogonal experiment is adopted in this work.

### 3.2. Orthogonal Experiment Results

The orthogonal experiment using four factors and three levels is adopted to plan the experimental scheme. Voltage, temperature, stirring speed, and time are selected as representative parameters. The orthogonal experimental design of the four factors and three levels, as shown in the first five columns in Table 5, is carried out to obtain the orthogonal calculation, while the last column lists the corresponding experimental results. The lowest surface roughness (Ra) is utilized as the index to investigate the electropolishing result by modifying the process parameters. Analyzing the results of the orthogonal test reveals that the minimum surface roughness in group 6 is 1.541 µm, which is the lowest value among all of the results. Table 6 displays the range analysis of the orthogonal experiment where $\bar{k_{1}}$, $\bar{k_{2}}$, and $\bar{k_{3}}$ refer to the mean values of level 1, 2, and 3, respectively.

**Figure 6:**
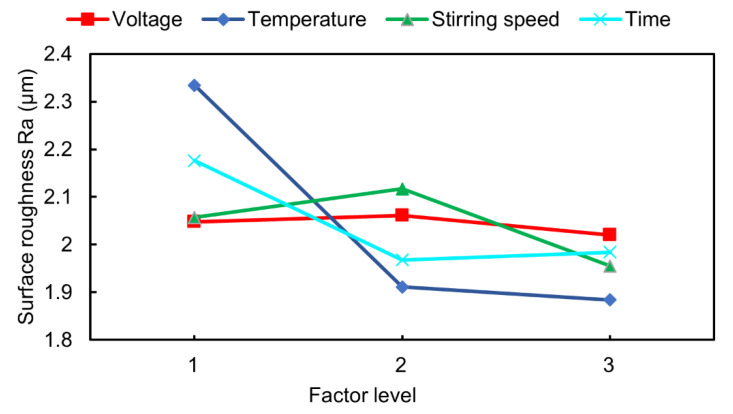
The influence trend of the factor levels on the internal surface roughness.

The analysis reveals that the influence of the three factors on the minimum surface roughness of the tubes’ internal surface follows the order of temperature, time, stirring speed, and voltage, with the temperature having the greatest impact and voltage having the smallest impact. Figure 6 illustrates the influence of the factors on the internal surface roughness. The temperature leads to a decrease in the internal surface roughness. At factor level 1 (343 K), the reaction products cannot be discharged because of the low flow speed. When the temperature increases, the ionic mobility in the inner flow field also increases. As a result, the conductivity increases and the polishing efficiency increases. With the higher temperature, the conductivity and the ion migration speed keep increasing, thus leading to a lower surface roughness. The time scheme initially shows a decrease followed by an increase, suggesting that excessively raising the polishing time may cause an increase in internal surface roughness due to over-polishing. 

In the early stage of electropolishing, surface materials are continuously removed, and the surface roughness decreases as the electrochemical reaction progresses. When the ideal polishing time is reached, the surface roughness achieves its optimal value. However, as the reaction continues, over-polishing occurs, leading to an increase in the surface roughness. Additionally, due to the large polished area, different positions inside may reach their optimal polishing state at different times; so, the ideal polishing time may fall within a range.

In addition, the voltage and stirring speed have an increase followed by a decrease. Unlike other studies, the effects of voltage and stirring speed on electropolishing performance are relatively smaller compared to other factors. This is mainly due to the different electrolytes used and the significantly larger polishing area (i.e., the whole internal surface in this work). Therefore, the increased polishing area results in a smaller improvement in the material removal rate per unit area with voltage change, thus reducing the impact of voltage on the surface roughness. On the other hand, the polishing rate is primarily influenced by the concentration of free ions in the electrolyte (governed by viscosity), rather than voltage. The stirring speed has little effect on altering the flow field, mainly due to the high viscosity of ethaline and the relatively small diameter of the tubes. Given the high viscosity of the electrolyte, increasing the stirring speed is likely less effective in improving the confined flow field limited by the tube structure. Hence, investigating the hydrodynamic conditions (i.e., considering viscosity or the electrolyte’s other fluid properties) inside the tube in the electropolishing process should be a potential avenue to optimize the internal surface electropolishing parameter combination.

**Figure 7:**
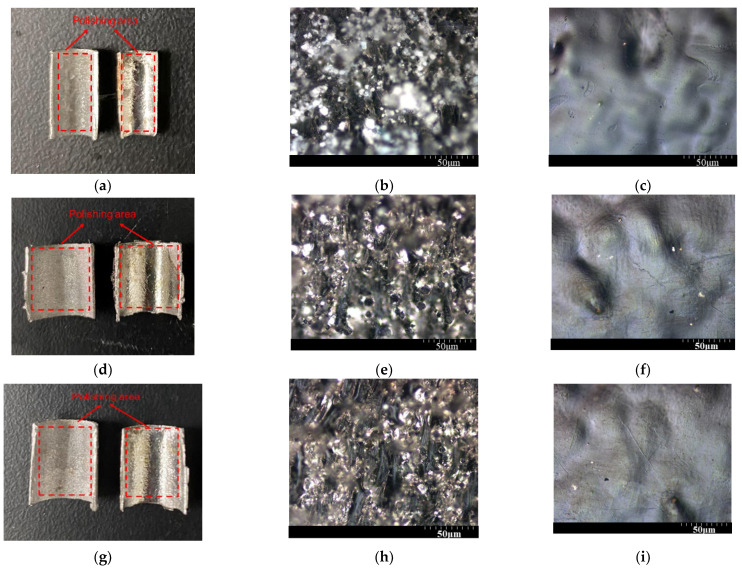
The polishing area of the Φ5 (**a**), Φ10 (**b**), and Φ8 mm (**c**) tubes and the internal surface image before (**d**,**e**,**h**) and after electropolishing (**g**,**f**,**i**) taken with a metallurgical microscope.

In summary, the optimal parameter combination to reduce the internal surface roughness is as follows: 353 K, 1800 s, 800 rpm, and 6 V. Then, Φ10, 8, and 5 mm tubes are polished with the optimal combination and the results are shown in Figure 7. In Figure 7a,d,g, the left section shows the initial surface, while the right section shows the polished surface. It can be observed in Figure 7b,e,h that the initial rough surface is covered with black particles formed by the spheroidization effect, along with adhered powder and noticeable unevenness. After electropolishing, according to Figure 7c,f,i, the surface flatness is significantly improved, with spherical particles and powder removed, and the surface becomes brighter, as shown in Figure 7a,d,g. However, compared to external/flat surface polishing, internal surface polishing is more prone to excessive local current density and elevated temperatures due to the significantly reduced gap between the electrodes and the restricted flow field area. Moreover, in the confined space filled with active Cl^-^ ions, pitting corrosion is observed. The internal surface roughness (Ra) of the Φ10, 8, and 5 mm tubes reduces from 8.419 μm, 7.684 μm, and 8.593 μm to 1.576 μm, 1.648 μm, and 1.535 μm (reduced by 81%, 78%, and 82%), respectively. For the Φ5 mm tube, the value of Ra reduces by 16.6% compared with the minimum Ra achieved in group 6 of the orthogonal experiment. The polished surfaces in Figure 7 exhibit a similar brightness and surface roughness in tubes with different diameters, indicating that ethaline performs uniformly across varying tube dimensions, effectively maintaining a consistent level of surface finish. Despite the pitting, ethaline is still a good electrolyte for tubes with various diameters.

**Figure 8:**
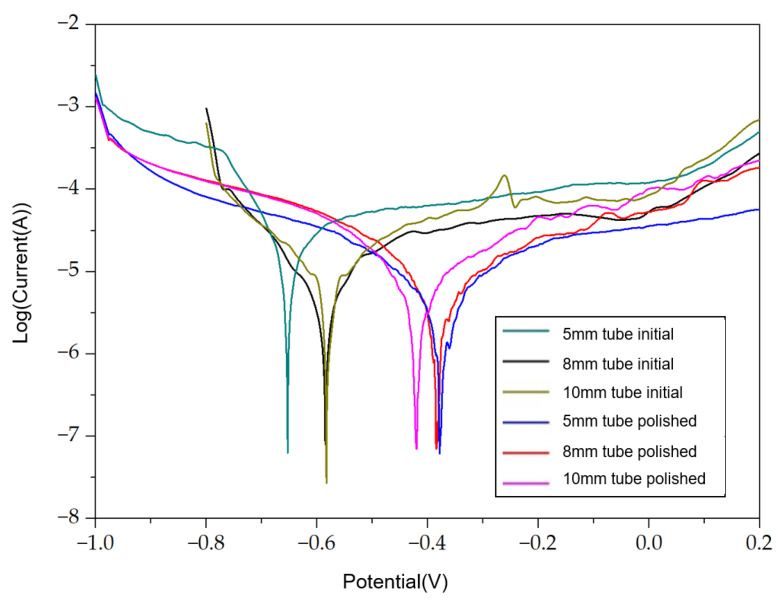
Tafel curves for short tubes.

Furthermore, the corrosion resistance of the internal surfaces of the tubes is quite important. It is essential to investigate whether the corrosion resistance is improved after polishing. The corrosion potentials and corrosion current values of the tubes before and after polishing are reported in Table 7. A significant increase in the polarization resistance of the tubes before and after polishing is found. The polarization resistance is almost six times the resistance before polishing. The Tafel curves shown in Figure 8 illustrate that the corrosion resistance of the tubes is improved after the ECP process.

### 3.3. Optimal Solution Application Experiment

**Figure 9:**
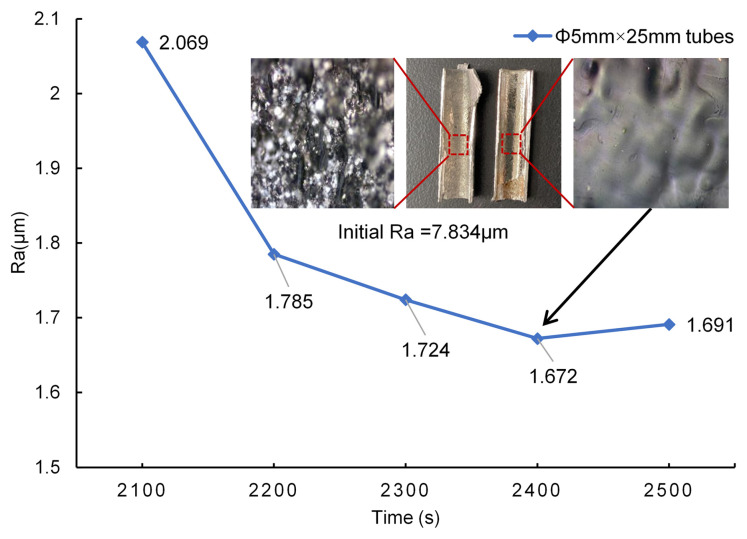
Ra vs. polishing time for electropolishing of long tubes.

The experiments described above focus on tubes with relatively small length-to-diameter ratios. In this section, the electropolishing results of using ethaline as an electrolyte for longer tubes (Φ5 mm × 25 mm tubes with a length–diameter ratio of 5) are presented. The optimal parameter combination is applied but the polishing time is increased because of the longer tubes and larger polishing area. Figure 9 shows the internal surface roughness change with polishing time.

The optimal parameter group is summarized in the previous part, showing that the surface roughness (Ra) is reduced from 7.834 μm to 1.672 μm (reduced by 78%). The internal surface has become as bright as that of short tube after polishing, as shown in Figure 7. In this section, it is shown that the polished surface shares a similar morphology to the short tube. The surface brightness in the middle section of the polished area of the long tube shows little difference compared to that of the short pipe. However, the surface brightness at the outlet of the long tube (i.e., the upper end of the long pipe in Figure 9) decreases due to the more turbulent flow field at the outlet, leading to reduced polishing consistency. The surface roughness (Ra) is approximately 9% higher than that of a shorter pipe with the same diameter. A larger length-to-diameter ratio may lead to a worse impact on internal surface quality.

**Figure 10:**
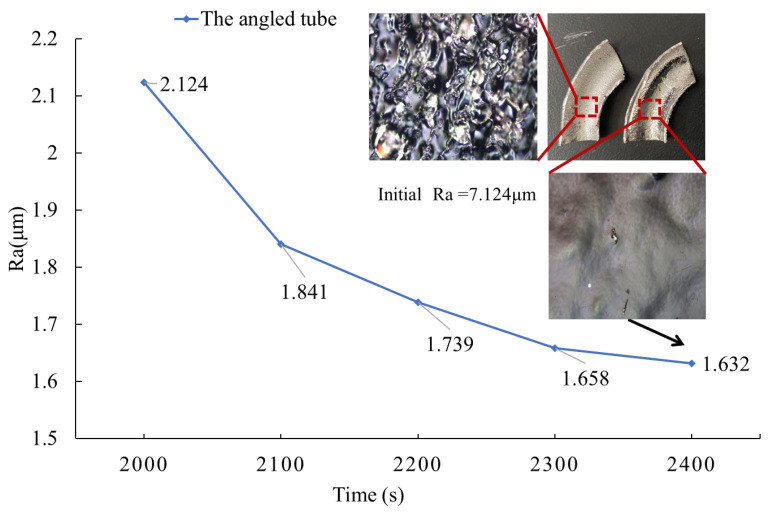
Ra vs. polishing time of angled tube polishing.

In industry applications, angled tubes are also widely used. However, internal surface polishing for angled tubes is much more difficult compared to the internal surface of straight tubes. First, the complex geometry of angled tubes may lead to polishing inconsistency. Second, due to the bending, the time for electrolytes to flow through the tubes is varied, which can also have a negative impact on polishing quality because of the concentration difference. In summary, the complex geometric structure manufactured by additive manufacturing leads to complicated physical fields inside the components. In the following section, the optimal parameter combination is applied to the angled tube polishing process to investigate whether ethaline still shows a good performance as it does in short tubes. As shown in Figure 10, unlike long tubes, the surface roughness keeps decreasing with time.

According to the figure which displays the comparison between the as-printed surface and the polished surface of the angled tube, the internal surface roughness (Ra) is reduced from 7.124 μm to 1.632 μm (reduced by 77%) and it shares a similar performance with the straight tubes. The brightness at the inner and outer diameters of the angled tube is lower compared to the middle section, but the brightness at the tube end (the lower end of the tube in Figure 10) is similar to that of the middle section, with no significant decrease. We believe this is due to the minimal effect of stirring on the flow field inside the bent tube since the orthogonal experimental results show that the stirring rate has a less noticeable impact on the polishing of the internal tube surface. It is noteworthy that some bulges which are not removed in the process still exist, as shown in Figure 10, while pitting is also found. It is considered that the cathode cannot match the curvature of the tubes perfectly so the current density does not keep consistent with other area. Furthermore, the magnetic stirrer generates a vortex which disturbs the flow field in the tubes. This indicates that the complex geometry makes it challenging to finish the internal surface of the angle tubes. As reported in the literature [20], the flow rate is an important factor for surface quality because the dissolution of the material in electropolishing is controlled by an adhesive layer which maintains a stable processing current. Therefore, with an inconsistent flow rate inside the angled tube, the bulges which are not removed completely appear more frequently than on the straight tubes. As the viscosity decreases, the flow rate will increase, and this leads to an unstable dissolution rate. Thus, selecting an electrolyte with relatively higher viscosity is a key factor in internal surface electropolishing.

### 3.4. Flow Field Simulation

To validate the advantages due to the high viscosity of electrolytes, in this section, a comparison is made between a NaCl electrolyte and ethaline by using COMSOL. The frozen rotor method is used to save time and calculation resources.

**Figure 11:**
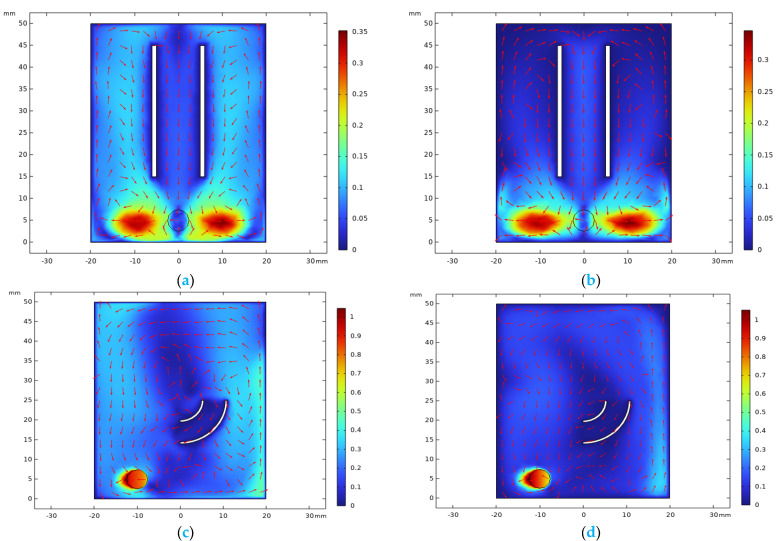
Velocity fields inside beaker: (**a**,**c**) NaCl solution; (**b**,**d**) ethaline.

Figure 11 displays the simulation results of the velocity distribution inside the reaction beaker. The two types of electrolyte flow fields share similarities. On one hand, at the bottom of the flow field, the stirrer drives the fluid outward radially, generating two latitudinal vortices, resulting in higher flow velocity around the bottom stirrer. On the other hand, the fluid flows rapidly along the bottom of the reaction vessel to the vessel wall and then forms a transient high-speed upward zone. This is due to the upward movement caused by the electrolyte moving radially, encountering the structural obstruction of the wall, and thus appearing as a short segment of the high-speed zone on the velocity map. In the case of DESs, the wall velocity increases briefly before dropping sharply, whereas for the NaCl solution, due to its lower viscosity, the wall velocity remains high and can be maintained up to the top region. Moreover, as observed from the flow velocity distribution in Figure 11, although the electrolyte inside the beaker undergoes circulation due to the action of the stirrer, the flow velocity of the electrolyte inside the angled tube is lower compared to outside the tube. Therefore, the effect of stirring on improving the electrolyte flow field within the angled tube is not so significant.

**Figure 12:**
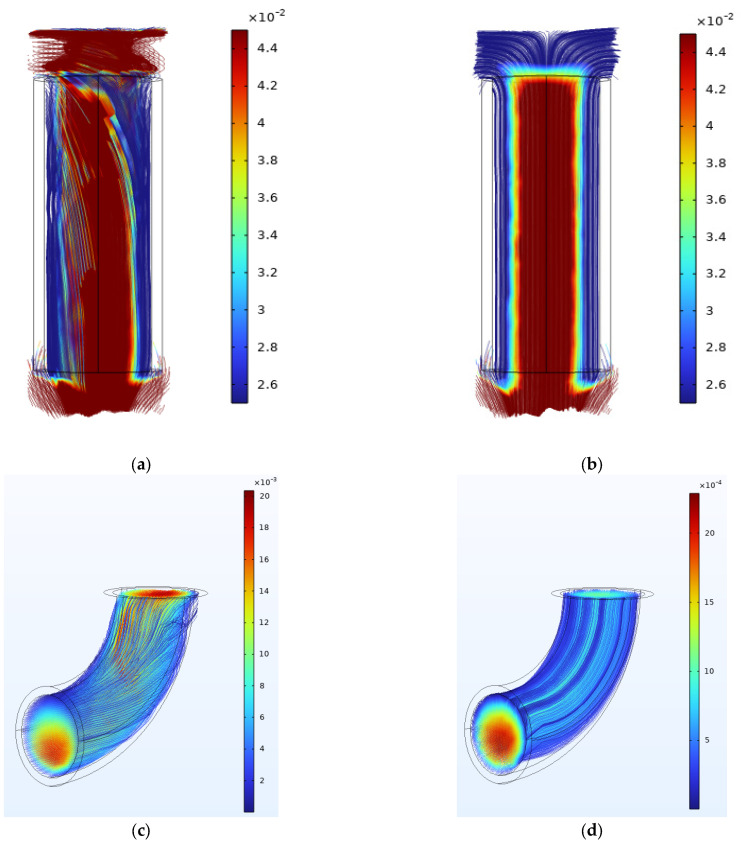
The streamline chart: (**a**,**c**) NaCl solution; (**b**,**d**) ethaline.

**Figure 13:**
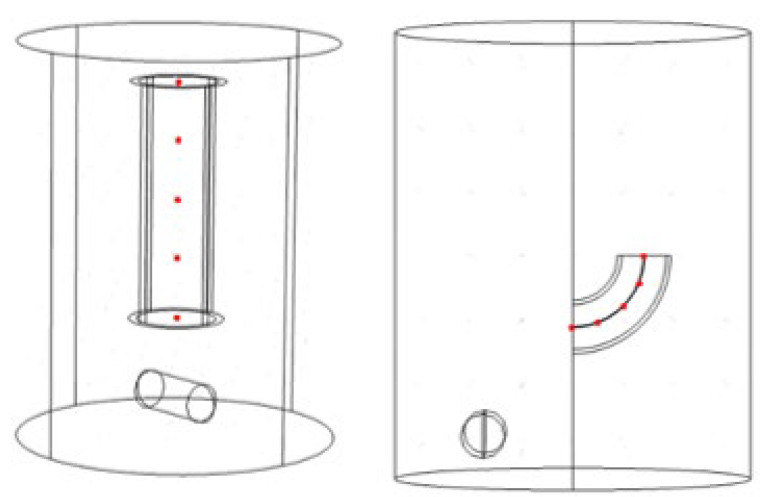
The point sketch map (red dots refer to the sampling points).

Figure 12 displays the detailed streamline inside the tubes in the NaCl solution and ethaline. The flow velocity is higher at the center of the parts and lower near the wall. For the NaCl solution, a vortex is found at the entrance of the components, whereas when ethaline is used as the electrolyte, the streamline is uniform. As discussed in the previous section, a vortex refers to an unstable dissolution rate, leading to deteriorating results. As discussed in the previous section, temperature has the greatest impact on polishing performance. The viscosity value is directly related to the temperature, suggesting that viscosity should also be taken into consideration in electropolishing for internal surfaces. To quantitatively describe the flow rate, five points are set with the same interval, as shown in Figure 13. The velocity at each point exported directly from COMSOL is listed in Table 8.

Table 8 shows that the velocities of both electrolytes at the first three points follow a pattern of initially decreasing and then increasing. This indicates that regardless of whether it is the high-viscosity ethaline or the low-viscosity NaCl solution, vortices are present when entering the pipe. However, due to the higher viscosity of ethaline, the change in velocity is not as noticeable. The standard deviation indicates that the flow velocity of ethaline is significantly less dispersed compared to that of the NaCl solution. On the other hand, in the angled tube, it is observed that the flow velocity is lowest at the bend (i.e., at the third point). Taking ethaline as an example, the flow velocity of ethaline in the bend decreases from 2.17 mm/s to 0.174 mm/s, with a reduction of approximately 92%. In actual processing, a low flow rate results in a low current density, which prevents further electropolishing. This explains the local polishing unevenness observed in the angled tube. 

The flow field simulation of hydrodynamic conditions provides a new optimization avenue of research for the electropolishing process [12]. In this section, we compared the flow field distribution in straight tubes and angled tubes. According to the simulation results, we validated that ethaline had a higher electropolishing consistency due to its high viscosity. Also, the complex streamline at the outlet of the tube in the simulation explains the reason why the brightness at the outlet is not as good as that of the middle section. This simulation indicates that hydrodynamic conditions should be taken into consideration when it comes to the electropolishing process with a small interelectrode gap or a restricted flow field area.

## 4. Conclusions

In this work, ethaline is adopted as the electrolyte for electropolishing the internal surfaces of straight and angled tubes composed of additive manufactured 316 L stainless steel. The orthogonal experiments are conducted to rank four factors’ impacts on the final surface roughness. The optimal factor combination is applied in further electropolishing experiments, and the flow field simulation is conducted. The following conclusions can be drawn:

In this study, we proposed an electropolishing method using ethaline for the internal surface of the tubes to reduce the surface roughness without any pre-treatment. The orthogonal experiments illustrate that temperature has the greatest impact on the surface roughness of the internal surfaces of 316 L tubes. For straight tubes with a diameter of 5 mm, 8 mm, and 10 mm, an optimal factor combination of 353 K, 1800 s, 800 rpm, and 6 V is applied and the surface roughness is reduced approximately by 82%, 78%, and 81%, respectively. For the Φ5 mm tube, the polarization resistance increases from 1465.7 Ω to 8329.8 Ω, nearly a sixfold improvement compared to the as-printed state.

The deep eutectic solvent, especially ethaline used in this work, indicates good processing repeatability when polishing the internal surface of 316 L tubes with a diameter of 5 mm and length-to-diameter ratios of 2 and 5; the surface roughness is 1.535 μm and 1.672 μm, respectively, using the same electrical parameters.

The flow field simulation illustrates that the viscosity of the electrolytes can also be a vital factor affecting polishing performance. However, the mechanism by which electrolyte viscosity affects polishing performance remains unclear and needs further research. Therefore, the simulation and modification of hydrodynamic conditions for a confined flow field of electropolishing can be a potential avenue of further research for the optimization of internal surface electropolishing processes.

## Figures and Tables

**Table 1:** SLM fabrication parameters.

Manufacturing Parameters	Value
Powder layer thickness	0.03 mm
Scanning speed	1250 mm/s
Laser power	500 W
Ambient temperature	150 °C

**Table 2:** Size of tubes.

Tube Type	Value
Short tube	Φ5 mm × 10 mm
Long tube	Φ5 mm × 25 mm
Angled tube diameter	8 mm
Angle	90°
Bending radius	13 mm

**Table 3:** Simulation parameters.

Simulation Parameter	Value
Stirring rate	800 rpm
Vessel height	50 mm
Vessel diameter	40 mm
Magnetic stirrer diameter	4 mm
Magnetic stirrer length	10 mm
Straight tube diameter	10 mm
Straight tube length	30 mm
Angled tube diameter	5 mm
Angled tube bending radius	8 mm
Ethaline viscosity	15 mPa·s
Ethaline density	1.085 kg/m^3^
NaCl solution viscosity	1.409 mPa·s
NaCl solution density	1.212 kg/m^3^

**Table 4:** Orthogonal experiment parameters of Φ5 mm tubes.

Level	Temperature (K)	Voltage (V)	Stirring Speed (rpm)	Time (s)
1	343	5	400	1600
2	348	6	600	1800
3	353	7	800	2000

**Table 5:** The orthogonal experimental results of the Φ5 mm tubes.

Group	Voltage (V)	Temperature (K)	Stirring Speed (rpm)	Time (s)	Ra (µm)
1	5	343	400	1600	2.486
2	5	348	600	1800	1.915
3	5	353	800	2000	1.742
4	6	343	600	2000	2.367
5	6	348	800	1600	1.974
6	6	353	400	1800	1.841
7	7	343	800	1800	2.149
8	7	348	400	2000	1.843
9	7	353	600	1600	2.069

**Table 6:** Range analysis.

Item	Parameters			
Surface Roughness Ra (µm)	Affected by Voltage	Affected by Temperature	Affected by Stirring Speed	Affected by Time
$\bar{k_{1}}$	2.048	2.334	2.057	2.176
$\bar{k_{2}}$	2.061	1.911	2.117	1.968
$\bar{k_{3}}$	2.02	1.884	1.955	1.984
Range	0.041	0.45	0.162	0.208
Rank	4	1	3	2

**Table 7:** Tafel curve data.

Group	$\beta_{a}/mV$	$\beta_{c}/mV$	$E_{corr}/mV$	$i_{corr}/\mu A\cdot {cm}^{2}$	$R_{P}/\Omega$
Φ10 mm initial	469.0	540.8	−0.583	12.18	3536.5
Φ10 mm polished	490.3	548.8	−0.378	5.023	8329.8
Φ8 mm initial	334.2	932.8	−0.585	7.664	4477.7
Φ8 mm polished	507.2	577.2	−0.384	5.706	7023.6
Φ5 mm initial	256.6	106.50	−0.654	22.45	1465.7
Φ5 mm polished	490.3	548.8	−0.378	5.023	8329.8

**Table 8:** Velocity distribution.

Sample Type	Solution	Point 1	Point 2	Point 3	Point 4	Point 5	Standard Deviation
Straight tube	Ethaline	31.8	18.6	19.2	18.8	15.8	5.62
	NaCl	102.8	36.7	51.1	47.7	31.0	25.5
Angled tube	Ethaline	2.17	0.863	0.174	0.754	0.109	0.6565
	NaCl	14.2	7.7	2.4	14.7	19.0	5.86

## Data Availability

The raw data supporting the conclusions of this article will be made available by the authors on request.
